# Tyrosol Suppresses Allergic Inflammation by Inhibiting the Activation of Phosphoinositide 3-Kinase in Mast Cells

**DOI:** 10.1371/journal.pone.0129829

**Published:** 2015-06-11

**Authors:** In-Gyu Je, Duk-Sil Kim, Sung-Wan Kim, Soyoung Lee, Hyun-Shik Lee, Eui Kyun Park, Dongwoo Khang, Sang-Hyun Kim

**Affiliations:** 1 Department of Pharmacology, School of Medicine, Kyungpook National University, Daegu 700–422, Republic of Korea; 2 Department of Thoracic and Cardiovascular Surgery, CHA Gumi Medical Center, CHA University, Gumi 730–040, Republic of Korea; 3 School of Life Sciences, Kyungpook National University, Daegu 702–701, Republic of Korea; 4 Department of Oral Pathology and Regenerative Medicine, School of Dentistry, Kyungpook National University, Daegu 700–412, Republic of Korea; 5 Department of Molecular Medicine, School of Medicine, Gachon University, Incheon 406–840, Republic of Korea; University of Kansas Medical Center, UNITED STATES

## Abstract

Allergic diseases such as atopic dermatitis, rhinitis, asthma, and anaphylaxis are attractive research areas. Tyrosol (2-(4-hydroxyphenyl)ethanol) is a polyphenolic compound with diverse biological activities. In this study, we investigated whether tyrosol has anti-allergic inflammatory effects. Ovalbumin-induced active systemic anaphylaxis and immunoglobulin E-mediated passive cutaneous anaphylaxis models were used for the immediate-type allergic responses. Oral administration of tyrosol reduced the allergic symptoms of hypothermia and pigmentation in both animal models. Mast cells that secrete allergic mediators are key regulators on allergic inflammation. Tyrosol dose-dependently decreased mast cell degranulation and expression of inflammatory cytokines. Intracellular calcium levels and activation of inhibitor of κB kinase (IKK) regulate cytokine expression and degranulation. Tyrosol blocked calcium influx and phosphorylation of the IKK complex. To define the molecular target for tyrosol, various signaling proteins involved in mast cell activation such as Lyn, Syk, phosphoinositide 3-kinase (PI3K), and Akt were examined. Our results showed that PI3K could be a molecular target for tyrosol in mast cells. Taken together, these findings indicated that tyrosol has anti-allergic inflammatory effects by inhibiting the degranulation of mast cells and expression of inflammatory cytokines; these effects are mediated via PI3K. Therefore, we expect tyrosol become a potential therapeutic candidate for allergic inflammatory disorders.

## Introduction

There are a range of allergic disorders including atopic dermatitis, allergic rhinitis, asthma, food allergy, and anaphylaxis. Mast cells are known to play key roles in these diseases through the production and secretion of allergic mediators; histamine, chemokines, cytokines, and growth factors [1]. Type 2 helper T (Th2) cells differentiated by stimulation of antigen-presenting cells activate B cells to produce immunoglobulin E (IgE), which binds to high affinity IgE receptor (FcεRI) on the surface of mast cells [2]. FcεRI-mediated mast cell activation is triggered by antigen-IgE cross-linking and leads to the degranulation and expression of inflammatory cytokines [3]. Mast cell signaling has been investigated thoroughly. Activation of Lyn and Syk causes phosphorylation of phosphoinositide 3-kinase (PI3K), which stimulates Akt and phospholipase C (PLC)γ [4]. Phosphorylation of the inhibitor of κB (IκB) kinase (IKK) complex by Akt and protein kinase C (PKC) results in activation of nuclear factor (NF)-κB and synaptosomal-associated protein (SNAP)23. In addition, PLCγ catalyzes the production of inositol 1,4,5-trisphosphate (IP_3_), which binds to IP_3_ receptors on the surface of the endoplasmic reticulum (ER). It causes release of calcium stored in the ER into the cytoplasm. Subsequently, the loss of calcium in the ER triggers a sudden increase of calcium influx from outside of the cell [5]. As a result, the expression and release of allergic molecules are enhanced by NF-κB, SNAP23, and increased intracellular calcium.

Histamine is the most important molecule in the acute allergy manifesting edema, warmth, and erythema by causing vasodilation, increasing vascular permeability, and leukocyte recruitment [6]. Inflammatory cytokines such as tumor necrosis factor (TNF)-α, interleukin (IL)-1β, and IL-4 lead to the chronic allergic phase by enhancing T cell activation or B cell survival [7]. Rat basophilic leukemia (RBL)-2H3 cells are suitable for *in vitro* studies of mast cell-mediated allergic inflammation involving the degranulation and expression of inflammatory cytokines [8]. Ovalbumin (OVA)-induced active systemic anaphylaxis (ASA) and IgE-mediated passive cutaneous anaphylaxis (PCA) are well-characterized animal models for immediate-type hypersensitivity [9,10].

Tyrosol (2-(4-hydroxyphenyl)ethanol), a polyphenolic compound contained in olive oil, has been reported to possess a wide range of biological activities including anti-oxidative, anti-apoptotic, and anti-inflammatory effects [11–13]. The present study compared the activities of tyrosol with gallic acid (3,4,5-trihydroxybenzoic acid) and dexamethasone already known for anti-allergic inflammatory properties [14,15]. In this study, we aimed to assess the effects of tyrosol on allergic inflammation using animal models for immediate-type hypersensitivity. In addition, anti-allergic effects related to the inhibition of mast cell degranulation and inflammatory cytokine expression was investigated using mast cells.

## Materials and Methods

### Animals

Male Imprinting Control Region (ICR) mice (aged 6 weeks) and Sprague-Dawley (SD) rats (aged 10 weeks) were purchased from the Dae-Han Biolink (Daejeon, Korea). Throughout the study, five animals per cage were housed in a room with laminar air flow, a temperature of 22 ± 2°C, and relative humidity of 55 ± 5%. Animal care and treatments were carried out in accordance with the guidelines established by the Public Health Service Policy on the Humane Care and Use of Laboratory Animals and were approved by the Institutional Animal Care and Use Committee of Kyungpook National University.

### Reagents and cell culture

Tyrosol (Figure A in S1 File), gallic acid, dexamethasone, anti-dinitrophenyl (DNP) IgE, DNP-human serum albumin (HSA), OVA, and Histodenz were purchased from Sigma-Aldrich (St. Louis, MO), and alum adjuvant was purchased from Thermo Scientific (Waltham, MA). RBL-2H3 cells and rat peritoneal mast cells (RPMCs) were grown at 37°C in 5% CO_2_ in Dulbecco’s modified Eagle’s medium and α-minimum essential medium (GIBCO, Grand Island, NY) respectively supplemented with 10% heat-inactivated fetal bovine serum (FBS), 100 U/ml penicillin G, 100 μg/ml streptomycin, and 250 ng/ml amphotericin B. RBL-2H3 cells were used throughout the study at a passage number ranging from 4 to 8.

### Preparation of RPMCs

Peritoneal cells were isolated from SD rats as previously described [10]. In brief, the rats were anesthetized with CO_2_ and injected with 20 ml of Tyrode’s buffer A (137 mM NaCl, 5.6 mM glucose, 12 mM NaHCO_3_, 2.7 mM KCl, 0.3 mM NaH_2_PO_4_, and 0.1% gelatin) into the peritoneal cavity, followed gentle massage of the abdomen for approximately 90 s. The peritoneal cavity was carefully opened, and the fluid containing the peritoneal cells was aspirated using a Pasteur pipette. The cells were collected after centrifugation at 150 *g* for 10 min at room temperature and then resuspended in 1 ml of Tyrode’s buffer A. This suspension was layered on 2 ml of 0.235 g/ml Histodenz solution and centrifuged at 400 *g* for 15 min at room temperature in order to separate the mast cells from other major components of rat peritoneal cells, i.e., macrophages and small lymphocytes. The cells at the buffer-Histodenz interface were discarded, and the cells in the pellet were washed and resuspended. Mast cell preparations had a purity of approximately 95%, based on toluidine blue staining. More than 97% of the cells were viable, based on trypan blue staining.

### OVA-induced ASA

Mice (*n* = 10/group) were sensitized with the OVA mixture (100 μg of OVA and 2 mg of alum adjuvant in 200 μl of phosphate-buffered saline [PBS]) by intraperitoneal injection on day 0 and day 7. Drugs including tyrosol, gallic acid, and dexamethasone were orally administered 3 times at doses of 0.1–10 mg/kg body weight (BW) (once every 2 days after the second sensitization). On day 14, 200 μg of OVA was intraperitoneally injected, and then the rectal temperature was measured every 10 min for 1 h. After 1 h, blood was obtained from the abdominal artery of each mouse for the measurement of serum histamine, OVA-specific IgE, and IL-4 levels.

### IgE-mediated PCA

An IgE-mediated PCA model was established as described previously [16]. To induce the PCA reaction, the skin on the ears of the mice (*n* = 5/group) was sensitized with an intradermal injection of anti-DNP IgE (0.5 μg/site) for 48 h. Drugs including tyrosol, gallic acid, and dexamethasone were orally administered at doses of 0.1–10 mg/kg BW 2 h before intravenous injection of DNP-HSA (1 mg/mouse) and 4% Evans blue (1:1) mixture. Thirty minutes later, the mice were euthanized, and both ears were collected to measure dye pigmentation. The amount of dye was determined colorimetrically after extraction with 0.5 ml of 1 M KOH and 4.5 ml of an acetone and phosphoric acid (5:13) mixture. The absorbance of the extract was measured using a spectrophotometer at a wavelength of 620 nm.

### Determination of mast cell degranulation

To determine mast cell degranulation, levels of histamine in serum and culture media were measured. Mouse blood was centrifuged at 400 *g* for 15 min at 4°C, and the serum was drawn. RBL-2H3 cells (5 × 10^5^/well in 12-well plates) were sensitized with anti-DNP IgE (50 ng/ml). After incubating overnight, the cells were pretreated with or without drugs including tyrosol, gallic acid, and dexamethasone for 1 h and then challenged with DNP-HSA (100 ng/ml) for 4 h. RPMCs (2 × 10^4^/well in 24-well plates) were handled similarly to RBL-2H3 cells; however, these were challenged with DNP-HSA for only 30 min. The cells were separated from the media by centrifugation at 150 *g* for 5 min at 4°C. To separate histamine from serum and media, 0.1 N HCl and 60% perchloric acid were added. After centrifugation, the supernatant fluid transferred to effendorf tube containing 5 N NaOH, 5 M NaCl, and *n*-butanol and vortexed. The organic phase was gathered, shaken with 0.1 N HCl and *n*-haptane, and then centrifuged. The histamine in the aqueous phase is assayed using the *o*-phthaldialdehyde spectrofluorometric procedure as previously described [17]. Fluorescent intensity was detected using a fluorescent plate reader (Molecular Devices) at an excitation wavelength of 360 nm and an emission wavelength of 440 nm. Release of β-hexosaminidase is also widely used as a marker for mast cell degranulation [16]. After incubation of media with β-hexosaminidase substrate buffer (100 mM sodium citrate, 1 mM 4-nitrophenyl *N*-acetyl-β-D-galactosaminide, pH 4.5) for 1 h at 37°C, the reaction was terminated using stop solution (0.1 M Na_2_CO_3_ and NaHCO_3_), and the absorbance was measured using a spectrophotometer at a wavelength of 405 nm.

### Determination of cell viability

Cell viability was determined by colorimetric analysis using 3-(4,5-dimethylthiazol-2-yl)-2,5-diphenyltetrazolium bromide (MTT) [18]. Water-soluble MTT is converted into water-insoluble formazan by mitochondrial dehydrogenase. RBL-2H3 cells (3 × 10^4^/well in 96-well plates) were pretreated with various concentrations of tyrosol for 24 h and incubated with 1 mg/ml MTT at 37°C. After 2 h, the formazan crystals were dissolved with DMSO, and then the absorbance was measured using a spectrophotometer (Molecular Devices, Sunnyvale, CA) at a wavelength of 570 nm. The absorbance of the formazan formed in untreated control cells was considered to represent 100% viability.

### RNA extraction and real-time PCR

Prior to isolation of total cellular RNA, RBL-2H3 cells (5 × 10^5^/well in 12-well plates) were sensitized with anti-DNP IgE (50 ng/ml). After incubating overnight, the cells were pretreated with or without drugs including tyrosol, gallic acid, and dexamethasone for 1 h and challenged with DNP-HSA (100 ng/ml) for 1 h. RNAiso Plus reagent (Takarabio Bio Inc., Shiga, Japan) was used to extract total RNA, in accordance with the manufacturer’s protocol. Complementary DNA (cDNA) was synthesized from 2 μg of total RNA using the Maxime RT-Pre Mix Kit (iNtRON Biotechnology, Daejeon, Korea). Quantitative real-time PCR was carried out using the Thermal Cycler Dice TP850 (Takarabio Bio Inc.) according to the manufacturer’s protocol. The total 25 μl reaction mixture was composed as follows: 1.5 μl of cDNA (150 ng), 1 μl of each of the forward and reverse primers (0.4 μM), 12.5 μl of SYBR Premix Ex Taq (Takarabio Bio Inc.), and 9 μl of dH_2_O. The conditions used for PCR were similar to those described previously [8].

### Enzyme-linked immunosorbent assay (ELISA)

The levels of inflammatory cytokines in media from RBL-2H3 cells challenged with DNP-HSA and serum were measured by ELISA [10]. ELISA was performed on a 96-well Nunc immuno plate using ELISA kits (BD Biosciences, San Diego, CA) according to the manufacturer’s protocol. Before the detection of OVA-specific IgE, the immune plate was coated with 20 μg of OVA instead of a capture antibody. After terminating the reaction of a substrate, the absorbance was measured using a spectrophotometer at a wavelength of 450 nm.

### Determination of the intracellular calcium level

Intracellular calcium was measured using the fluorescent indicator Fluo-3/AM (Invitrogen, Carlsbad, CA) [10]. RBL-2H3 cells (3 × 10^4^/well in 96-well plates) were sensitized with anti-DNP IgE (50 ng/ml). After incubating overnight, the cells were preincubated with Fluo-3/AM for 1 h at 37°C and washed with Tyrode’s buffer B (137 mM NaCl, 5.5 mM glucose, 12 mM NaHCO_3_, 2.7 mM KCl, 0.2 mM NaH_2_PO_4_, 1 mM MgCl_2_, and 1.8 mM CaCl_2_) to remove the dye from the cell surface. The cells were pretreated with or without tyrosol for 1 h and then challenged with DNP-HSA (100 ng/ml). BAPTA-AM (Calbiochem, La jolla, CA), a calcium chelator, was used as a positive control. Fluorescent intensity was detected using a fluorescent plate reader at an excitation wavelength of 485 nm and an emission wavelength of 510 nm. The intracellular calcium level in untreated control cells was assigned a relative absorbance value of 1.

### Western blot

Nuclear and cytosolic proteins were extracted as previously described [16]. Before protein extraction, RBL-2H3 cells (2 × 10^6^/well in 6-well plates) were sensitized with anti-DNP IgE (50 ng/ml). After incubating overnight, the cells were pretreated with or without tyrosol for 1 h and challenged with DNP-HSA (100 ng/ml). PP2 (Calbiochem), an inhibitor of Src family kinases, was used as a positive control. After suspension in 100 μl of cell lysis buffer A (0.5% Triton X-100, 150 mM NaCl, 10 mM HEPES, 1 mM EDTA/Na_3_VO_4_, 0.5 mM PMSF/DTT, and 5 μg/ml leupeptin/aprotinin), the cells were vortexed, incubated for 5 min on ice, and centrifuged at 400 *g* for 5 min at 4°C. The supernatant was collected and used as the cytosolic protein extract. The pellets were washed 3 times with 1 ml of PBS and then suspended in 25 μl of cell lysis buffer B (25% glycerol, 420 mM NaCl, 20 mM HEPES, 1.2 mM MgCl_2_, 0.2 mM EDTA, 1 mM Na_3_VO_4_, 0.5 mM PMSF/DTT, and 5 μg/ml leupeptin/aprotinin), vortexed, sonicated for 30 s, incubated for 20 min on ice, and centrifuged at 15,000 *g* for 15 min at 4°C. The supernatant was collected and used as the nuclear protein extract. Proteins were separated using 8–12% sodium dodecyl sulfate-polyacrylamide gel electrophoresis and transferred to a nitrocellulose membrane. Immunodetection was carried out using a chemiluminescent substrate (Thermo Scientific). The following antibodies were purchased from Santa Cruz Biotech (Santa Cruz, CA); phospho-IKK (sc-21661, Ser^176^, goat polyclonal, 1:1000), IKK (sc-7607, rabbit polyclonal, 1:1000), NF-κB (sc-109, rabbit polyclonal, 1:1000), IκBα (sc-371, rabbit polyclonal, 1:1000), actin (sc-8432, mouse monoclonal, 1:1000). The following antibodies were purchased from Cell signaling Technology (Beverly, MA); phospho-Lyn (#2731, Tyr^507^, rabbit polyclonal, 1:1000), phospho-Syk (#2711, Tyr^525/526^, rabbit polyclonal, 1:1000), phospho-PI3K (#4228, Tyr^458^, rabbit polyclonal, 1:1000), phospho-Akt (#9271, Ser^473^, rabbit polyclonal, 1:1000), Lyn (#2732, rabbit polyclonal, 1:1000), Syk (#2712, rabbit polyclonal, 1:1000), PI3K (#4292, rabbit polyclonal, 1:1000), Akt (#9272, rabbit polyclonal, 1:1000).

### Statistical analysis

Each data presented as a graph represents the mean ± standard error (SE) of independent experiments. Statistical analyses were performed using Prism5 (GraphPad Software, San Diego, CA), and treatment effects were analyzed using a one-way analysis of variance followed by Dunnett’s test. A value of *P* < 0.05 was used to indicate a significant difference.

## Results

### Effects of tyrosol on systemic and local anaphylaxis

**Fig 1:**
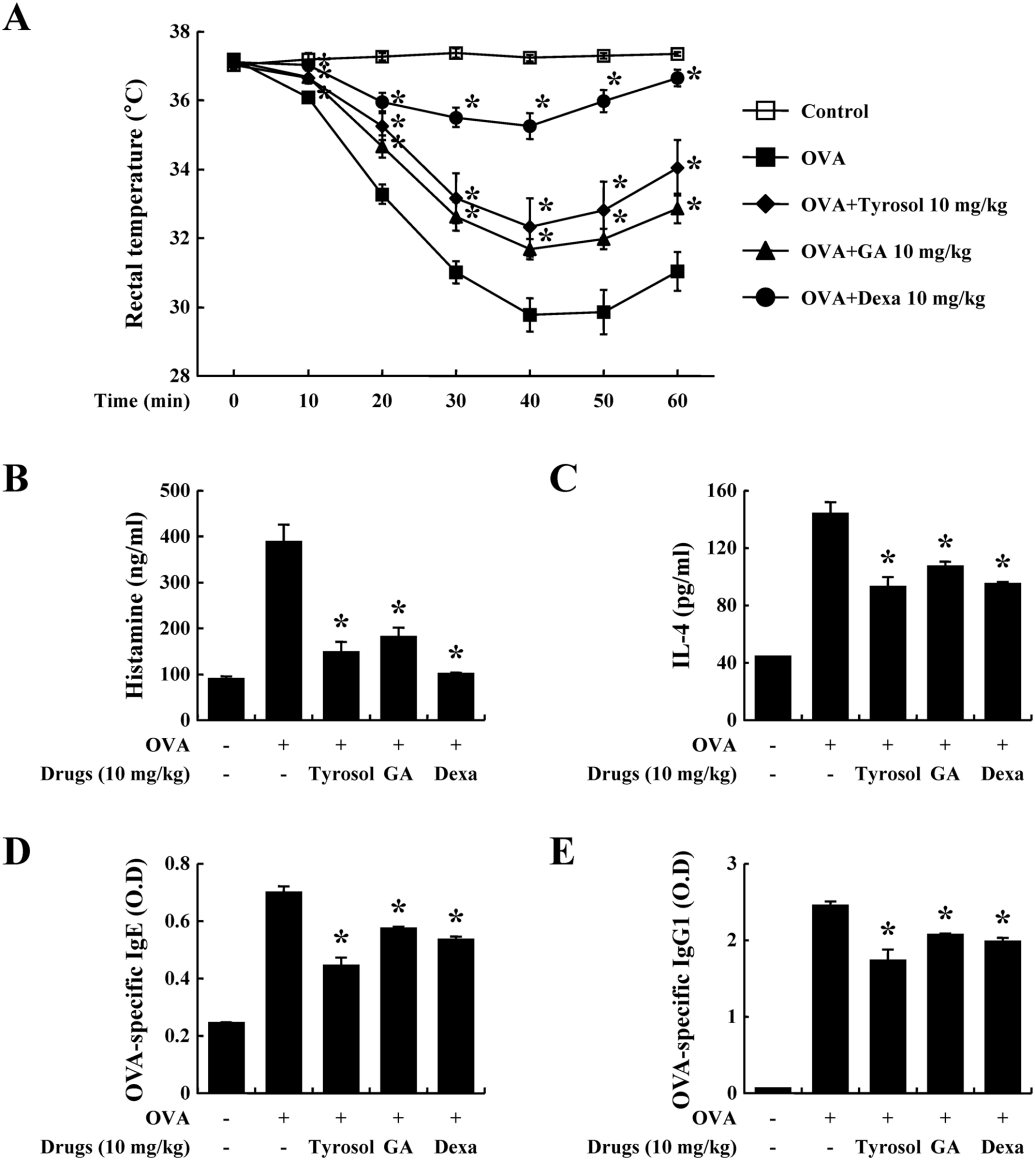
Effects of tyrosol on ovalbumin-induced active systemic anaphylaxis. Induction of systemic anaphylaxis and oral administration of drugs including tyrosol, GA, and Dexa were described in the Materials and methods section. (A) Rectal temperature was measured every 10 min for 1 h. Blood was obtained from the abdominal artery of each mouse for the measurement of serum histamine, OVA-specific IgE, and IL-4 levels. (B) Histamine level was assayed using the *o*-phthaldialdehyde spectrofluorometric procedure. (C-E) IL-4, OVA-specific IgE, and OVA-specific IgG1 levels were measured by ELISA. Each data presented as a graph represents the mean ± SE (*n* = 10 per group). *Significant difference from OVA-challenged group at *P* < 0.05. GA: gallic acid; Dexa: dexamethasone.

The OVA-induced systemic anaphylaxis model is appropriate for the investigation of the anti-allergic inflammatory effects of drug candidates [9]. After challenges of OVA, mice repetitively sensitized with OVA and alum adjuvant developed anaphylaxis and were monitored for 1 h. The rectal temperature of the mice decreased over 40–50 min; administration of tyrosol alleviated this hypothermia, which was associated with the serum histamine level (Fig 1A). The serum histamine level increased by about 4 times and was also diminished by tyrosol (Fig 1B). In addition, tyrosol attenuated the serum IL-4 level (Fig 1C). IL-4 is a representative Th2 cytokine, that plays important role in inducing IgE synthesis [19]. Binding of IgE and IgG1 with FcεRI is necessary for the activation of mast cells and allergic responses, especially anaphylaxis [20]. We measured OVA-specific IgE and IgG1 levels, which were considerably increased by sensitizations of OVA (Fig 1D and 1E). Tyrosol showed more effective inhibition in the generation of both immunoglobulins than did gallic acid and dexamethasone.

**Fig 2:**
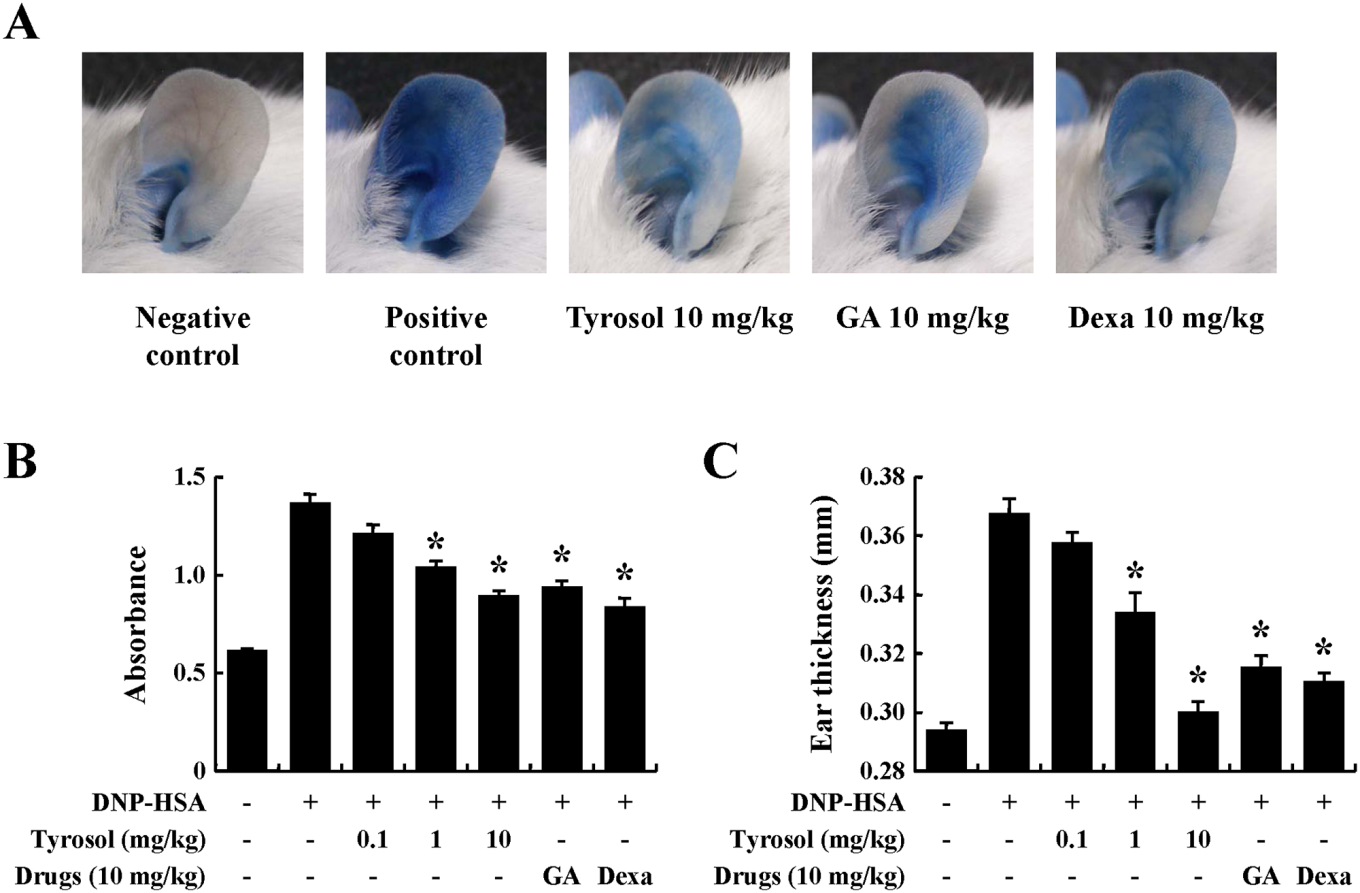
Effects of tyrosol on IgE-mediated passive cutaneous anaphylaxis. Mouse ear skin (*n* = 5/group) was sensitized with an intradermal injection of anti-DNP IgE (0.5 μg/site) for 48 h. Drugs including tyrosol, GA, and Dexa were orally administered at doses of 0.1–10 mg/kg BW 2 h before the intravenous injection of DNP-HSA (1 mg/mouse) and 4% Evans blue (1:1) mixture. Thirty minutes later, the thickness of both ears was measured, and the ears were collected to measure pigmentation. Dye was extracted as described in the Materials and methods section and detected using a spectrophotometer. Each data presented as a graph represents the mean ± SE (*n* = 5 per group). *Significant difference from DNP-HSA challenged group at *P* < 0.05. GA: gallic acid; Dexa: dexamethasone.

PCA is another widely used animal model for immediate-type allergic reactions [16]. After challenges of antigen, a blue spot developed at the sensitized site because of the increased vascular permeability caused by histamine released from mast cells. Tyrosol decreased the size and color of the blue spot in a dose-dependent manner (Fig 2A and 2B). Increased vascular permeability induced ear swelling, which was also reduced by tyrosol (Fig 2C).

### Effects of tyrosol on the mast cell degranulation and inflammatory cytokine expression

**Fig 3:**
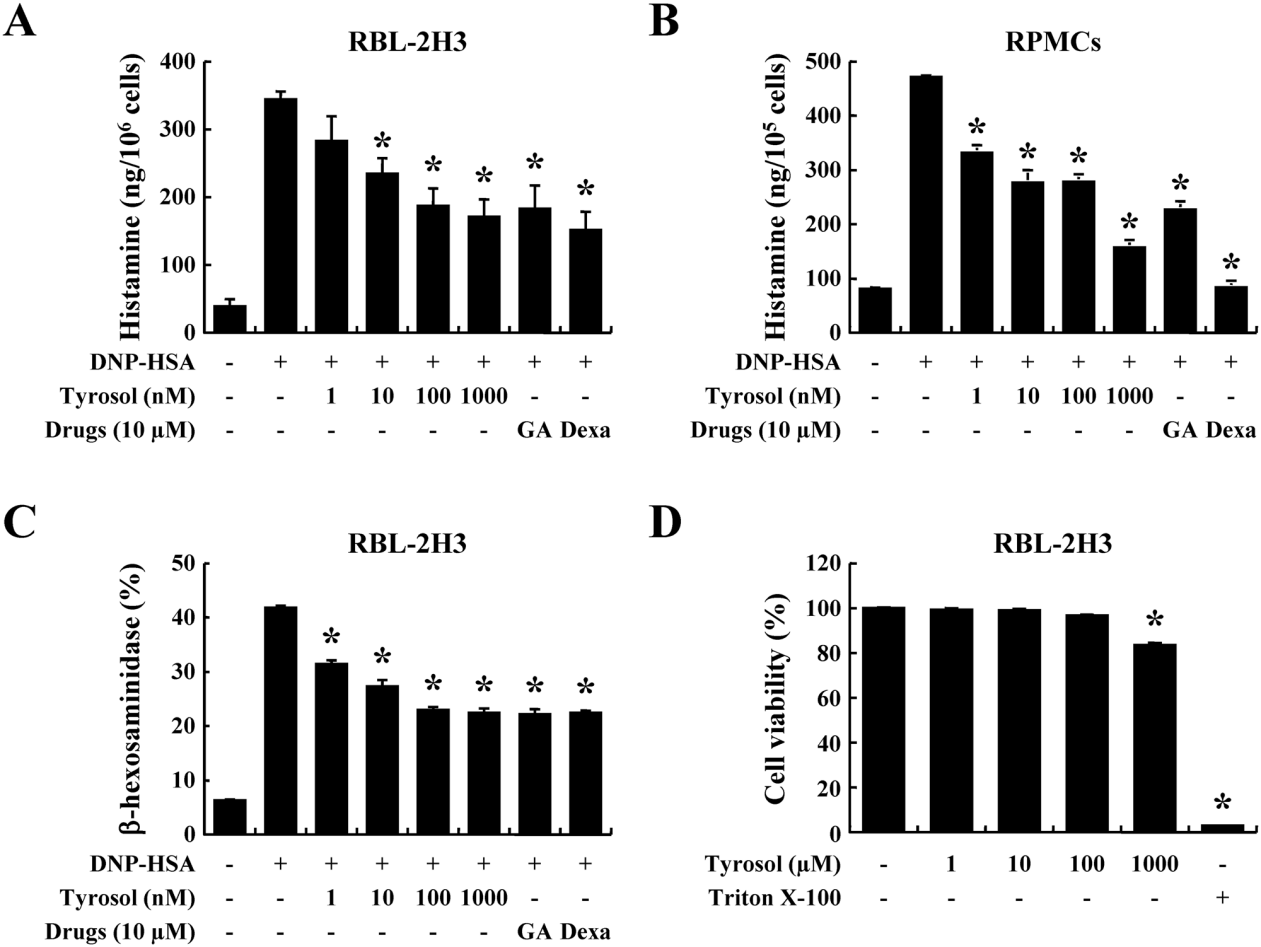
Effects of tyrosol on mast cell degranulation. (A, B) RBL-2H3 cells (5 × 10^5^/well) and RPMCs (2 × 10^4^/well) were sensitized with anti-DNP IgE (50 ng/ml). After incubating overnight, the cells were pretreated with or without drugs including tyrosol, GA, and Dexa for 1 h and then challenged with DNP-HSA (100 ng/ml). Histamine level was assayed using the *o*-phthaldialdehyde spectrofluorometric procedure. (C) The level of β-hexosaminidase was measured using β-hexosaminidase substrate buffer. (D) RBL-2H3 cells (3 × 10^4^/well) were pretreated with or without tyrosol for 24 h and then incubated with 1 mg/ml MTT for 2 h. The absorbance intensity was detected using a spectrophotometer. Each data presented as a graph represents the mean ± SE of three independent experiments. *Significant difference from DNP-HSA challenged group at *P* < 0.05. GA: gallic acid; Dexa: dexamethasone.

Mast cells are major producers of histamine, a key molecule in allergic responses [6]. Therefore, inhibition of mast cell degranulation is a useful therapeutic target for the treatment of allergic symptoms. To examine the influence of tyrosol on the degranulation of mast cells, we measured histamine release in RBL-2H3 cells and RPMCs. Histamine was rapidly released after challenges of antigen, however this release was dose-dependently hindered by tyrosol in both mast cells (Fig 3A and 3B). Tyrosol showed a similar or superior inhibitory effect in comparison with gallic acid and dexamethasone although at 10 times lower concentration. The β-hexosaminidase assay commonly used to evaluate mast cell degranulation was carried out [16]. Suppressive effect of tyrosol presented an equal aspect in both secretion of histamine and β-hexosaminidase (Fig 3C). MTT assays were performed to investigate the cytotoxicity of tyrosol. Tyrosol did not manifest any significant cytotoxicity following 24 h exposure to concentration of up to 100 μM (Fig 3D). These results indicated that tyrosol reduces mast cell degranulation in the absence of cytotoxicity.

**Fig 4:**
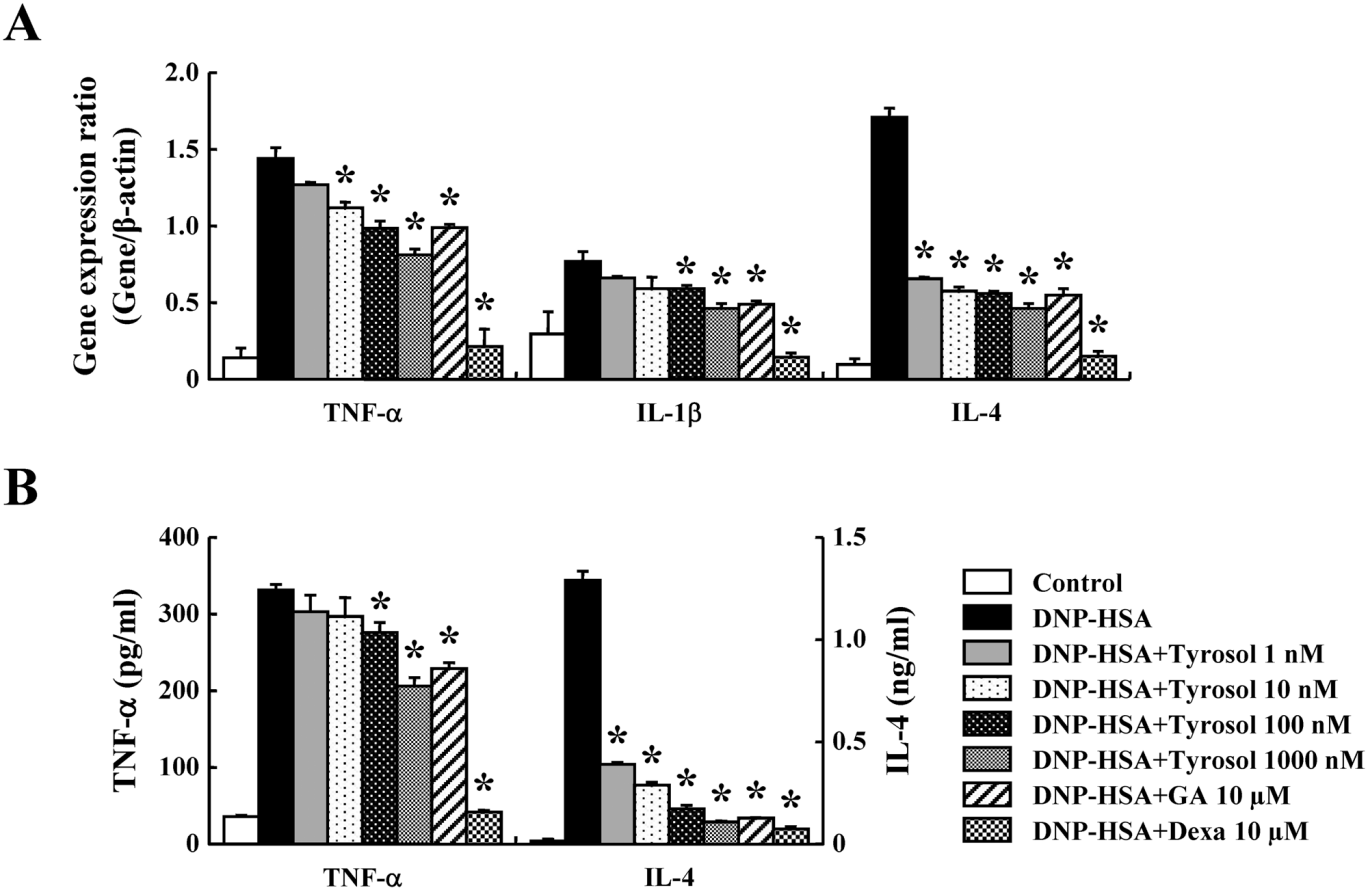
Effects of tyrosol on inflammatory cytokine expression. (A) RBL-2H3 cells (5 × 10^5^/well) were sensitized with anti-DNP IgE (50 ng/ml). After incubating overnight, the cells were pretreated with or without drugs including tyrosol, GA, and Dexa for 1 h and then challenged with DNP-HSA (100 ng/ml). Extraction and analysis of mRNA were performed as described in the Materials and methods section. The gene expression of inflammatory cytokines was determined by real-time PCR. (B) The secretion of inflammatory cytokines was measured by ELISA. Each data presented as a graph represents the mean ± SE of three independent experiments. *Significant difference from DNP-HSA challenged group at *P* < 0.05. GA: gallic acid; Dexa: dexamethasone.

Inflammatory cytokines are known to mediate inflammation by enhancing recruitment and activation of immune cells [21]. To assure the effects of tyrosol on the expression of inflammatory cytokines such as TNF-α, IL-1β, and IL-4 in RBL-2H3 cells, real-time PCR and ELISAs were carried out. The expression of these cytokines was elevated by activation of FcεRI, while it was suppressed by tyrosol dose-dependently; these effects were observed at the mRNA and protein levels (Fig 4A and 4B).

### Effects of tyrosol on intracellular calcium and activation of NF-κB in mast cells

**Fig 5:**
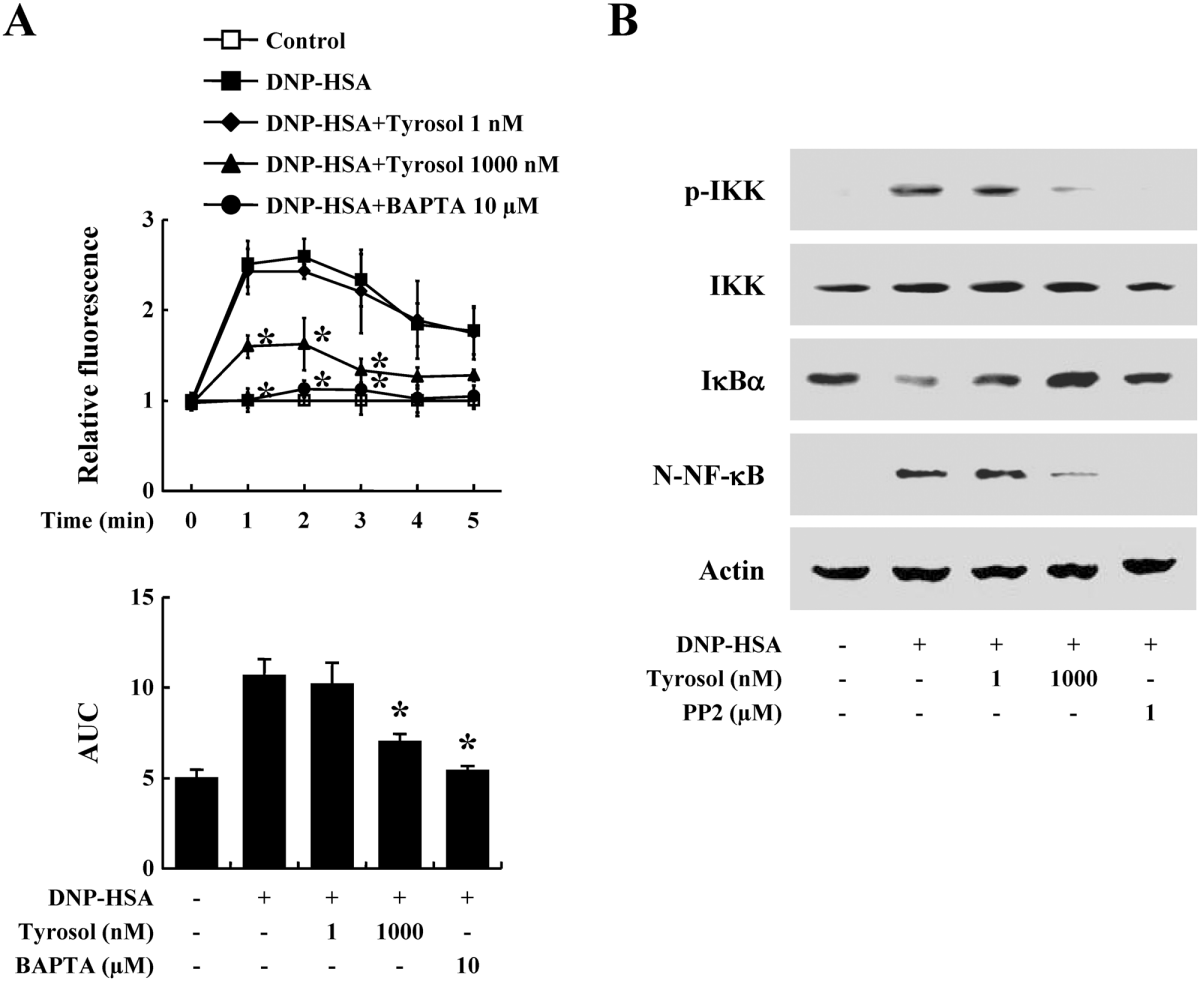
Effects of tyrosol on intracellular calcium and the NF-κB activation in mast cells. (A) RBL-2H3 cells (3 × 10^4^/well) were sensitized with anti-DNP IgE (50 ng/ml). After incubating overnight, the cells were preincubated with Fluo-3/AM for 1 h. The cells were pretreated with or without tyrosol for 1 h and then challenged with DNP-HSA (100 ng/ml). Intracellular calcium was detected every 1 min for 5 min using a fluorescent plate reader. Area under the curve (AUC) was calculated over 5 min. BAPTA, a calcium chelator, was used as a positive control. Each data presented as a graph represents the mean ± SE of three independent experiments. *Significant difference from DNP-HSA challenged group at *P* < 0.05. (B) RBL-2H3 cells (2 × 10^6^/well) were sensitized with anti-DNP IgE (50 ng/ml). After incubating overnight, the cells were pretreated with or without tyrosol for 1 h and then challenged with DNP-HSA (100 ng/ml). Activations of IKK complex and NF-κB were assayed by Western blot (N-: nuclear, p-: phosphorylated). The bands of actin and total form were used as a loading control. PP2, a Src family inhibitor, was used as a positive control. The band is a representative of three independent experiments.

Calcium is an important secondary messenger in mast cell signaling. The intracellular calcium level increased by activation of FcεRI leads to mast cell degranulation and expression of inflammatory cytokines [22]. PLCγ generates IP_3_, which binds to IP_3_ receptor and triggers calcium efflux from the ER; the inflow of extracellular calcium is enhanced by this ER calcium depletion [5]. In the present study, the intracellular calcium level rapidly increased within a few seconds of exposure to DNP-HSA, however tyrosol reduced this rise of intracellular calcium level (Fig 5A).

NF-κB is a major transcription factor regulating the expression of inflammatory cytokines. Degradation of IκBα allows the movement of NF-κB into the nucleus. The IKK complex activates NF-κB by phosphorylating IκBα [4]. The IKK complex has 3 subunits; α, β, and regulatory subunits. Recent research has reported that the IKKβ subunit activates FcεRI-induced exocytosis. Thus, inhibition of the IKK complex might suppress degranulation and inflammatory cytokine expression in mast cells. Our results showed that tyrosol hindered activation of NF-κB and the IKK complex (Fig 5B).

### Effects of tyrosol on the activation of signaling proteins in mast cells

**Fig 6:**
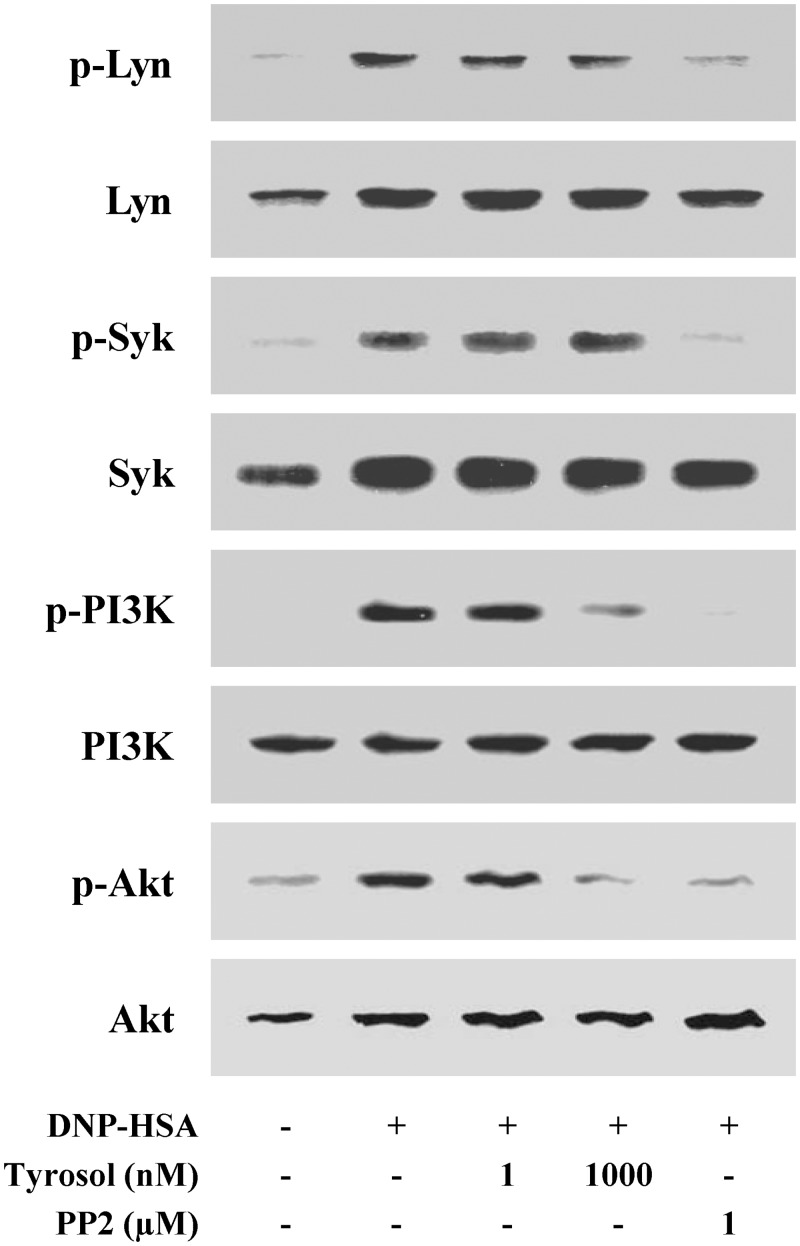
Effects of tyrosol on the activation of signaling proteins. RBL-2H3 cells (2 × 10^6^/well) were sensitized with anti-DNP IgE (50 ng/ml). After incubating overnight, the cells were pretreated with or without tyrosol for 1 h and then challenged with DNP-HSA (100 ng/ml). Extraction of protein was performed as described in the Materials and methods section. Activation of signaling proteins was assayed by Western blot (p-: phosphorylated). The band of total form was used as a loading control. PP2, a Src family inhibitor, was used as a positive control. The band is a representative of three independent experiments.

The intracellular signaling pathways in mast cells have been determined in detail [4]. To assure the phosphorylation of signaling proteins such as Lyn, Syk, PI3K, and Akt, Western blots were performed. Tyrosol did not affect the activation of Lyn and Syk, on the contrary, it reduced the phosphorylation of PI3K and Akt (Fig 6).

To confirm that inhibition of PI3K regulates degranulation and cytokine expression in mast cells, we used NVP-BEZ235, an inhibitor of PI3K/Akt [23]. NVP-BEZ235 certainly worked as a PI3K inhibitor in RBL-2H3 cells (Figure B in S1 File). The release of histamine and β-hexosaminidase was suppressed by blocking the activation of PI3K (Figure C and D in S1 File). In addition, FcεRI-induced expression of TNF-α and IL-4 was completely blocked by NVP-BEZ235 (Figure E and F in S1 File). According to these results, it seems reasonable to conclude that the inhibition of PI3K by tyrosol reduces allergic responses by suppressing mast cell degranulation and cytokine expression.

## Discussion

Anaphylaxis induced by the rapid release of allergic mediators such as histamine, heparin, and various cytokines can have lethal consequences. OVA-induced ASA and IgE-mediated PCA are suitable animal models for immediate-type hypersensitivity. Mice sensitized by OVA and alum adjuvant developed hypothermia after challenges of OVA because this produced a sudden increase in the serum histamine level. IgE and IgG levels significantly contribute to systemic anaphylaxis in mice [24]. IL-4, a principal Th2 cytokine, differentiates naive T cells into Th2 cells, which stimulate production of IgE in B cells [2]. It is likely that tyrosol reduces hypothermia by inhibiting histamine release from mast cells and lessening production of IgE and IgG resulted from decreasing IL-4 levels in mice. We observed that tyrosol more effectively hindered production of IgE and IgG1 than gallic acid and dexamethasone. The tyrosol-mediated reduction in pigmentation observed in PCA is also considered to arise from its suppression of mast cell degranulation. Histamine released from mast cells enhances vascular permeability. Consequently, intravascular circulating Evans blue percolates through the endodermis. These results indicated that tyrosol has therapeutic effects on anaphylactic reaction.

Mast cells are important for the manifestation of IgE-mediated allergic responses by releasing various mediators such as histamine, lipid-derived mediators, chemokines, cytokines, and growth factors [1]. Therefore, mast cells are key targets for the development of medicines for allergic disorders. To assure the anti-allergic inflammatory effects of tyrosol on mast cells, RBL-2H3 cells and RPMCs were used in this study. We assessed the release of histamine and β-hexosaminidase to measure mast cell degranulation. Histamine from mast cells causes typical allergic symptoms such as edema, warmth, and erythema [6]. Thus, attenuating mast cell degranulation is a viable therapeutic strategy for the treatment of allergic disorders. Tyrosol dose-dependently decreased degranulation without cytotoxic effects.

Inflammatory cytokines such as TNF-α, IL-1β, and IL-4 play crucial roles in prompting and sustaining chronic allergy. TNF-α promotes adaptive immunity through the activation of NF-κB and stimulates the migration, maturation, and differentiation of immune cells [25,26]. IL-1β aggravates auto-inflammatory and allergic diseases such as contact hypersensitivity, atopic dermatitis, and bronchial asthma [27]. IL-4 is necessary for allergic responses as it drives the generation of IgE in plasma B cells [19]. Our results showed that expression of inflammatory cytokines was increased after activation of FcεRI and this effect was dose-dependently reduced by tyrosol in RBL-2H3 cells. In the previous research, tyrosol suppressed expression of TNF-α in an anoxia-induced EAhy926, human endothelial cell line [28]. Furthermore, production of TNF-α, IL-1β, and IL-6 was attenuated on peripheral blood mononuclear cells and RAW 264.7 cells stimulated with lipopolysaccharide [13,29]. These reports support the present research, which assure that tyrosol reduced cytokine expression. As a result, it is possible that tyrosol-mediated blockade of inflammatory cytokines expression alleviates allergic inflammation and limits progression in chronic allergy.

Calcium is a crucial secondary messenger in mast cell signaling [5]. The intracellular calcium level regulates exocytosis from mast cells and also expression of inflammatory cytokines [30–32]. In our results, the intracellular calcium level in RBL-2H3 cells stimulated with DNP-IgE was rapidly elevated, while tyrosol blocked calcium influx. Activation of NF-κB is important for the expression of various inflammatory cytokines including TNF-α, IL-1β, and IL-4 [33]. Phosphorylation of IκBα prompts its proteolytic degradation, which allows NF-κB to translocate into the nucleus. FcεRI-stimulated degradation of IκBα and nuclear translocation of NF-κB were regulated by tyrosol. The IKK complex induces activation of NF-κB [4]. Tyrosol also reduced phosphorylation of IKK, and this seemed to obstruct the degradation of IκBα and nuclear translocation of NF-κB. A recent study informed the relationship between IKK and mast cell degranulation. IKKβ stimulated SNAP-23 which has a critical role in mast cell degranulation and anaphylactic responses [34]. We predicted that suppression of the IKK complex might improve allergic inflammatory reactions by lessening degranulation and the expression of allergic mediators. It is likely that tyrosol-related inhibition of degranulation and cytokine expression is associated with the reduction of intracellular calcium level and IKK activation in mast cells, since NF-κB plays is a major transcription factor regulating expression of inflammatory cytokine.

Signaling pathways of mast cells have been extensively studied [4]. Lyn, Syk, PI3K, and Akt were selected as the representative signaling proteins to anticipate the target of tyrosol. Our results indicated that tyrosol interrupted the protein phosphorylation from PI3K without affecting Lyn and Syk. Blockade of PI3K reduces activation of PLCγ, which regulates PKC and the intracellular calcium level [35]. PLCγ hydrolyzes phosphatidylinositol 4,5-bisphosphate (PIP_2_) to diacylglycerol (DAG) and IP_3_ [36]. DAG is reported to activate PKC, which stimulates the IKK complex; IP_3_ ultimately triggers extracellular calcium influx. These IKK stimulation and increased intracellular calcium level provoke the secretion and expression of allergic mediators. The Akt dependent- pathway also activates the IKK complex [4]. PI3K produces phosphatidylinositol 3,4,5-trisphosphate, an important lipid mediator of Akt activation, from PIP_2_. Accordingly, it is possible that the suppression of PI3K regulates the IKK complex by interrupting the activation of not only PLCγ but also Akt. These studies are consistent with our expectation that tyrosol reduces allergic inflammation by inhibiting the activation of PI3K. Nevertheless, we only estimate that the target point of tyrosol is between Syk and PI3K. Further studies are required to identify the exact target proteins of tyrosol.

In the present study, we aimed to demonstrate the effects of tyrosol on allergic inflammation using animal models and mast cells. Tyrosol attenuated anaphylactic shock in immediate-type hypersensitivity models, and this effect is related to a decreased release of allergic mediators from mast cells. Secretion and expression of allergic molecules are decreased by tyrosol in a dose-dependent manner, and we anticipate that the anti-allergic inflammatory effects of tyrosol are associated with inhibition of PI3K, which regulates activation of the IKK complex and the intracellular calcium level in mast cells. Further studies are needed in order to determine the exact binding target of tyrosol. Nevertheless, this study suggests that tyrosol is a possible therapeutic candidate for allergic disorders by inhibiting degranulation and the expression of inflammatory cytokines in mast cells.

## Supporting Information

S1 File Effects of NVP-BEZ235 on mast cell degranulation and inflammatory cytokine expression. (Figure A) Chemical structure of tyrosol. (Figure B) RBL-2H3 cells (2 × 10^6^/well) were sensitized with anti-DNP IgE (50 ng/ml). After incubating overnight, the cells were pretreated with or without NVP-BEZ235, an inhibitor of PI3K, for 1 h and then challenged with DNP-HSA (100 ng/ml). Extraction of protein was performed as described in the Materials and methods section. Activation of PI3K was assayed by Western blot (p-: phosphorylated). The band of total form was used as a loading control. PP2, a Src family inhibitor, was used as a positive control. The band is a representative of three independent experiments. (Figure C) RBL-2H3 cells (5 × 10^5^/well) were sensitized with anti-DNP IgE (50 ng/ml). After incubating overnight, the cells were pretreated with or without drugs including NVP-BEZ235, GA, and Dexa for 1 h and then challenged with DNP-HSA (100 ng/ml). Histamine level was assayed using the *o*-phthaldialdehyde spectrofluorometric procedure. (Figure D) The level of β-hexosaminidase was measured using β-hexosaminidase substrate buffer. (Figure E and F) The secretion of inflammatory cytokines was measured by ELISA. Each data presented as a graph represents the mean ± SE of three independent experiments. *Significant difference from DNP-HSA challenged group at *P* < 0.05. GA: gallic acid; Dexa: dexamethasone.
(TIF)

S2 File The action schema of tyrosol in mast cells. Tyrosol blocked the IgE-mediated phosphorylation of PI3K. Blockade of PI3K reduces activation of Akt and downstream IKK complex. Decrease of IKK and intracellular calcium results in the reduction of secretion of allergic mediators.
(TIF)

S1 Archive All the images are the original Western blot data for Fig 5B, Fig 6, and Figure B in S1 File. (ZIP)
