# Supplementary figures and images for: Tyrosol Suppresses Allergic Inflammation by Inhibiting the Activation of Phosphoinositide 3-Kinase in Mast Cells
Source: PLoS One. 2015 Jun 11;10(6):e0129829. doi: 10.1371/journal.pone.0129829 (PMC4465982; doi:10.1371/journal.pone.0129829)

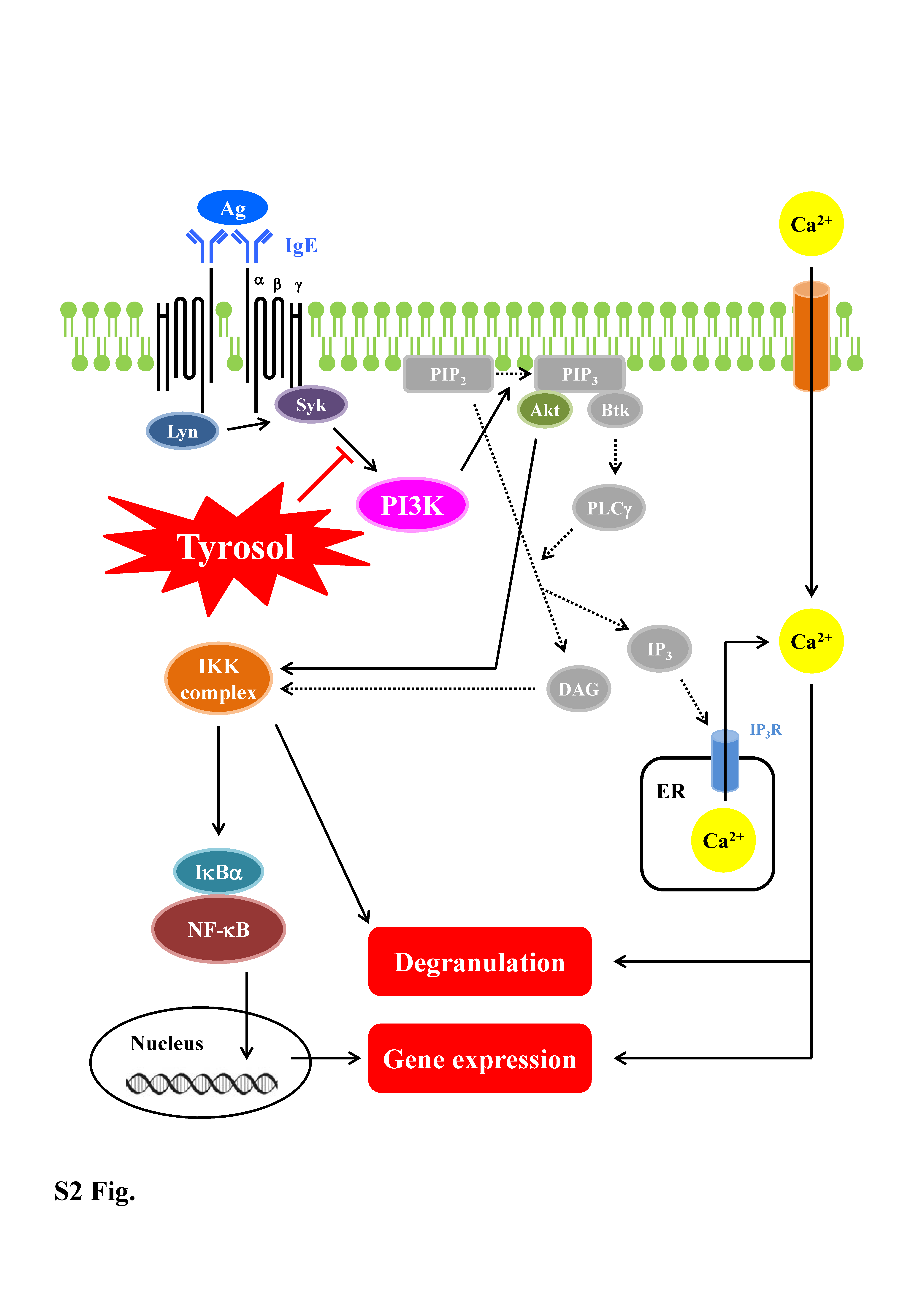

Supplement: S2 File — Tyrosol blocked the IgE-mediated phosphorylation of PI3K. Blockade of PI3K reduces activation of Akt and downstream IKK complex. Decrease of IKK and intracellular calcium results in the reduction of secretion of allergic mediators. (TIF) [file pone.0129829.s002.tif]

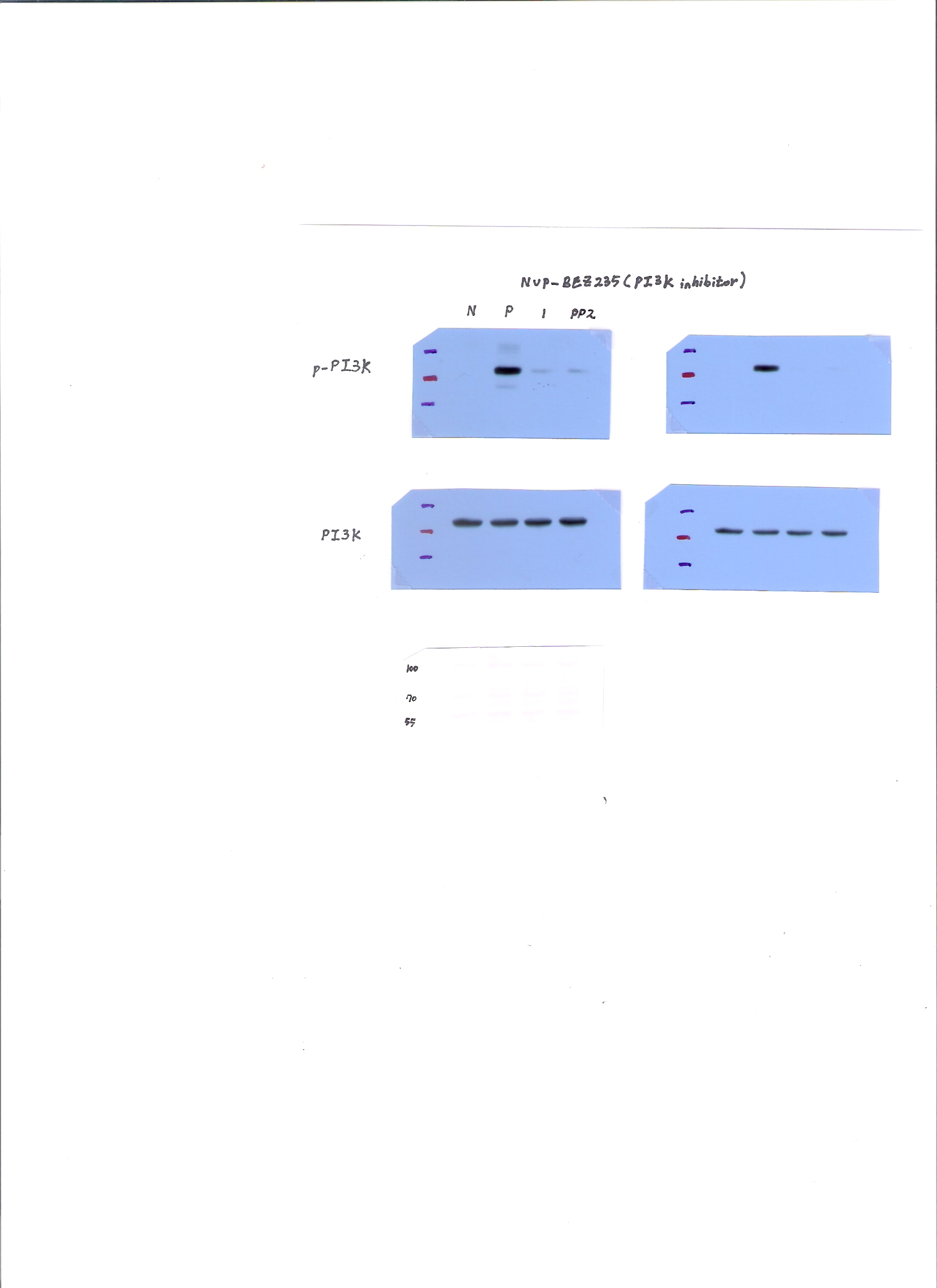

Supplement: S1 Archive — (ZIP) [file pone.0129829.s003.zip › PI3KI PI3K-original.jpg]

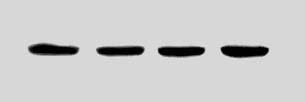

Supplement: S1 Archive — (ZIP) [file pone.0129829.s003.zip › PI3KI PI3K.jpg]

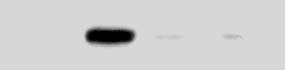

Supplement: S1 Archive — (ZIP) [file pone.0129829.s003.zip › PI3KI p-PI3K.jpg]

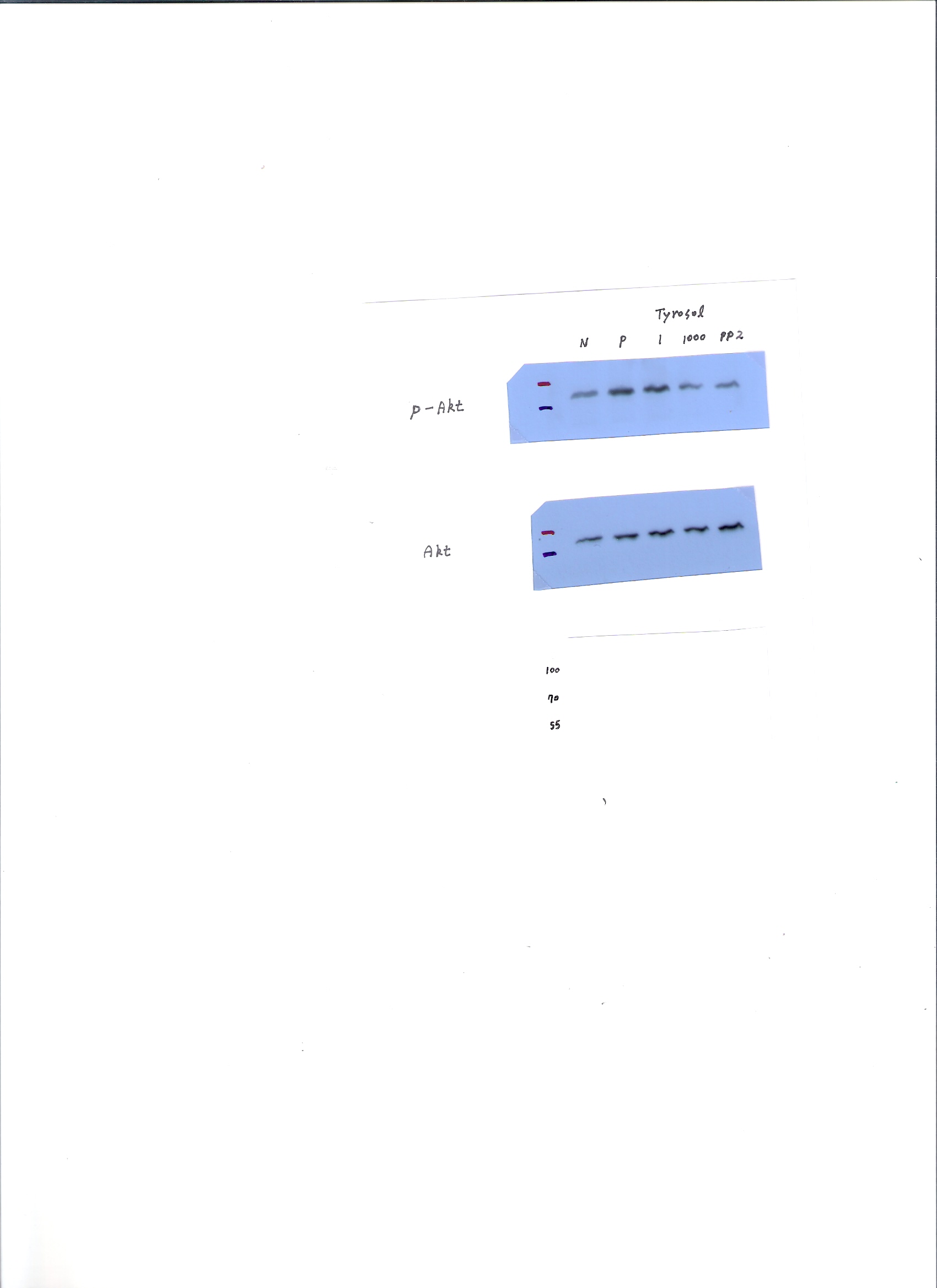

Supplement: S1 Archive — (ZIP) [file pone.0129829.s003.zip › Tyrosol Akt-original.jpg]

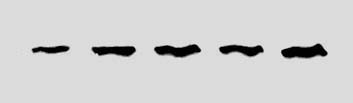

Supplement: S1 Archive — (ZIP) [file pone.0129829.s003.zip › Tyrosol Akt.jpg]

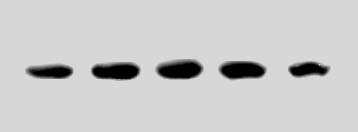

Supplement: S1 Archive — (ZIP) [file pone.0129829.s003.zip › Tyrosol IKK.jpg]

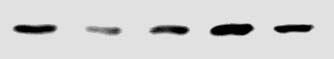

Supplement: S1 Archive — (ZIP) [file pone.0129829.s003.zip › Tyrosol IkBa.jpg]

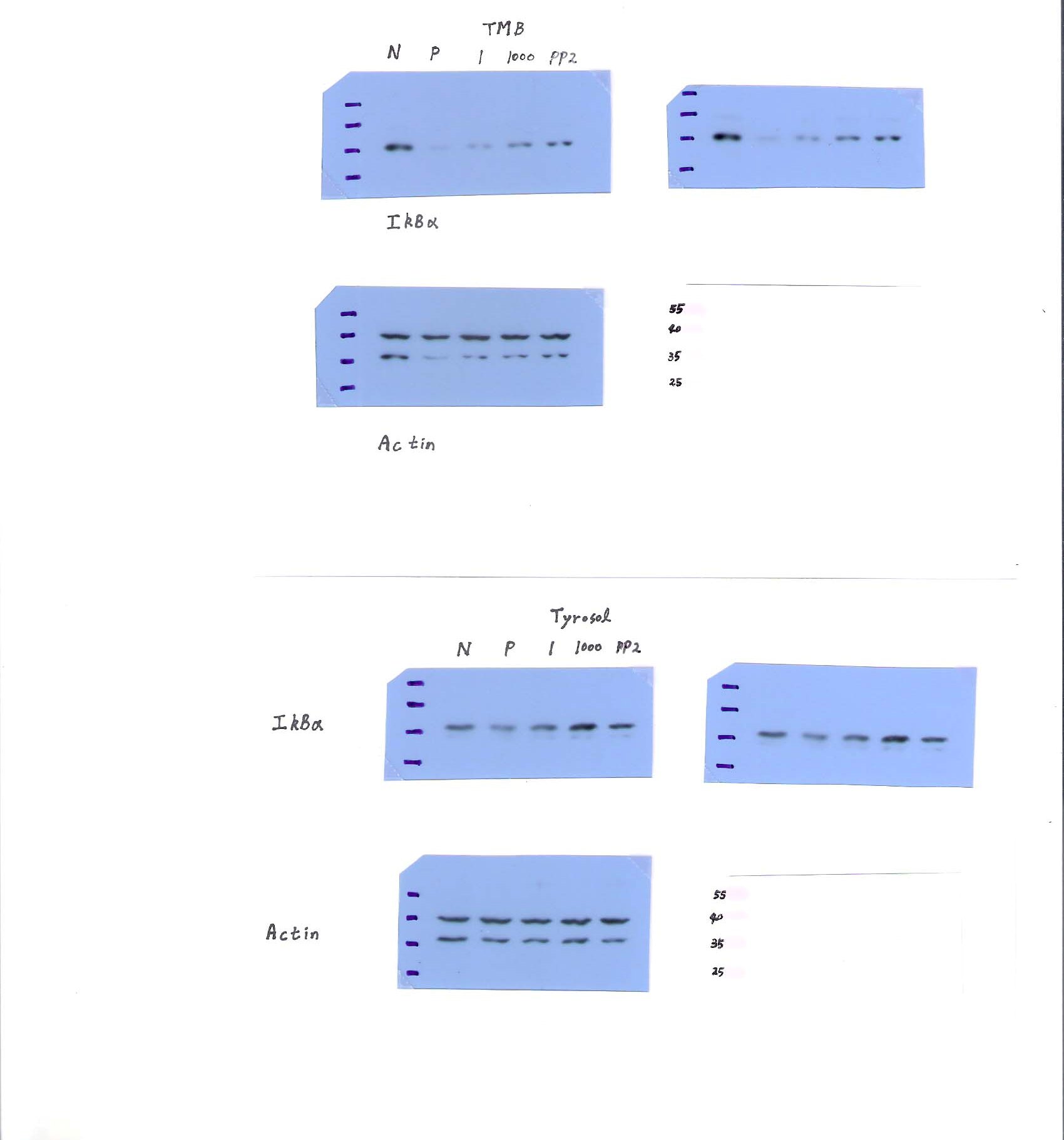

Supplement: S1 Archive — (ZIP) [file pone.0129829.s003.zip › Tyrosol IÑΩBÑß, actin-original.jpg]

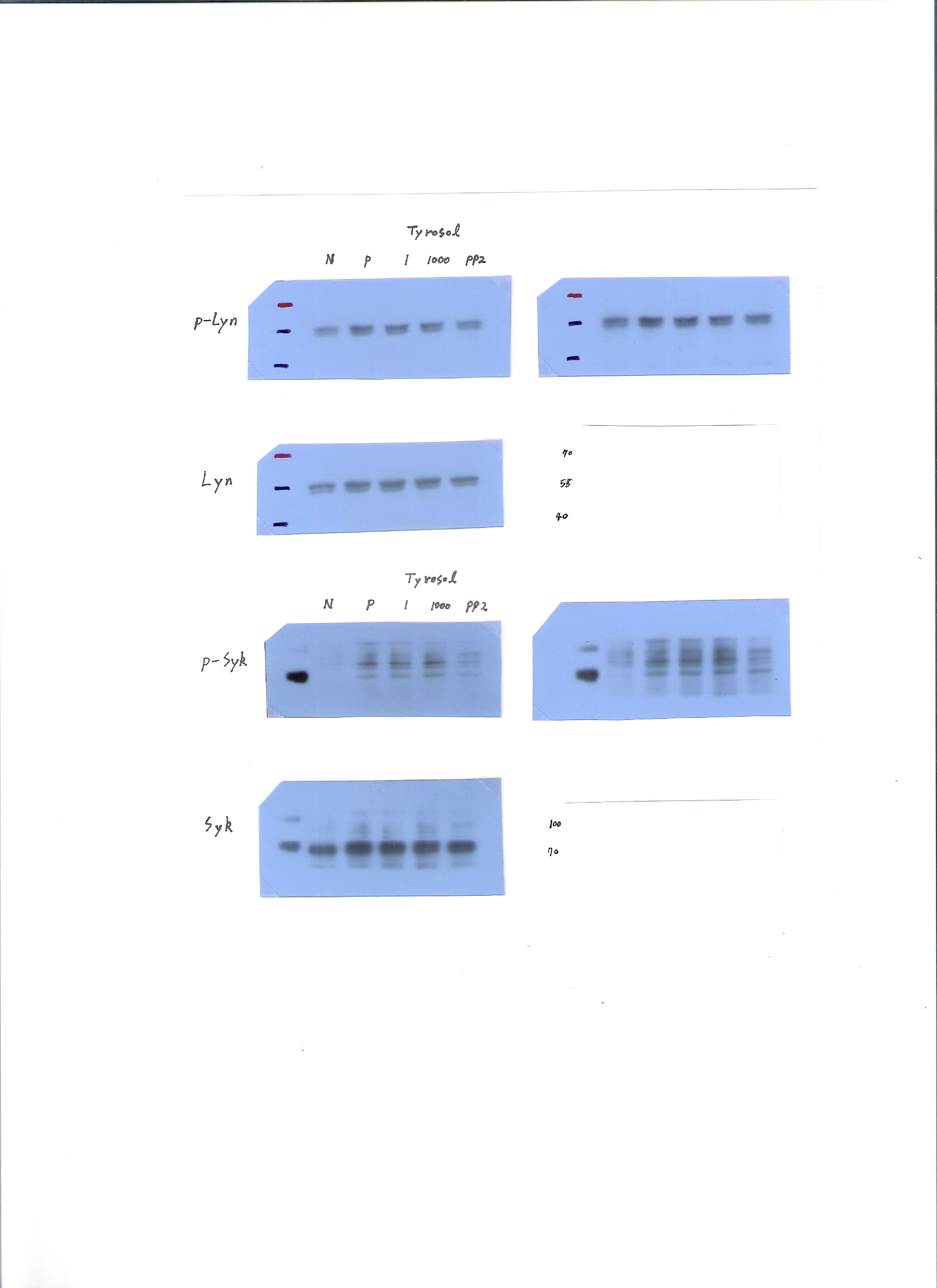

Supplement: S1 Archive — (ZIP) [file pone.0129829.s003.zip › Tyrosol Lyn, Syk-original.jpg]

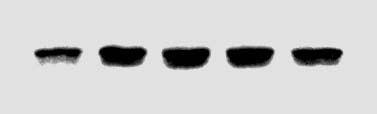

Supplement: S1 Archive — (ZIP) [file pone.0129829.s003.zip › Tyrosol Lyn.jpg]

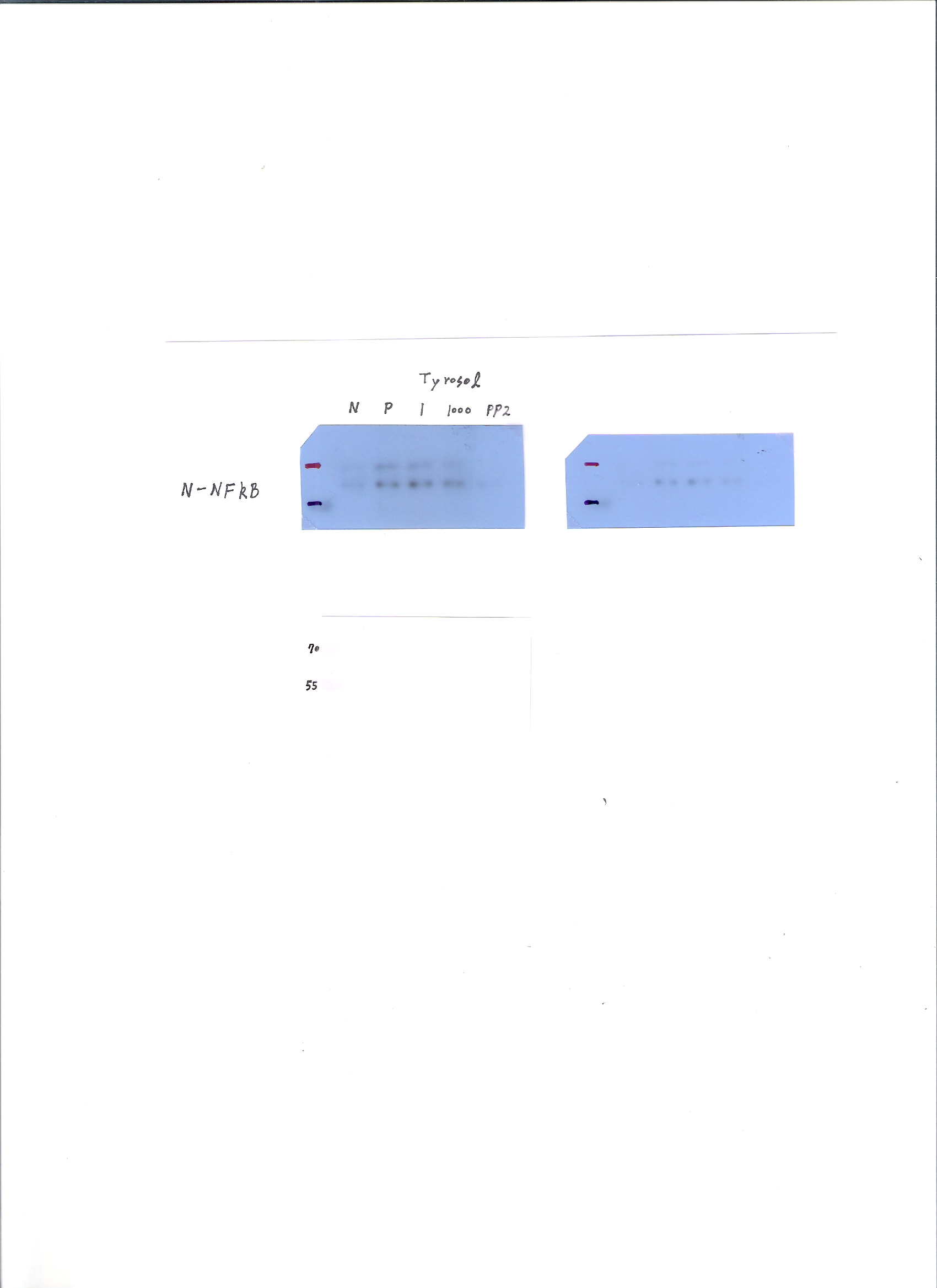

Supplement: S1 Archive — (ZIP) [file pone.0129829.s003.zip › Tyrosol NF-ÑΩB-original.jpg]

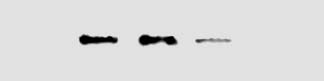

Supplement: S1 Archive — (ZIP) [file pone.0129829.s003.zip › Tyrosol NF-ÑΩB.jpg]

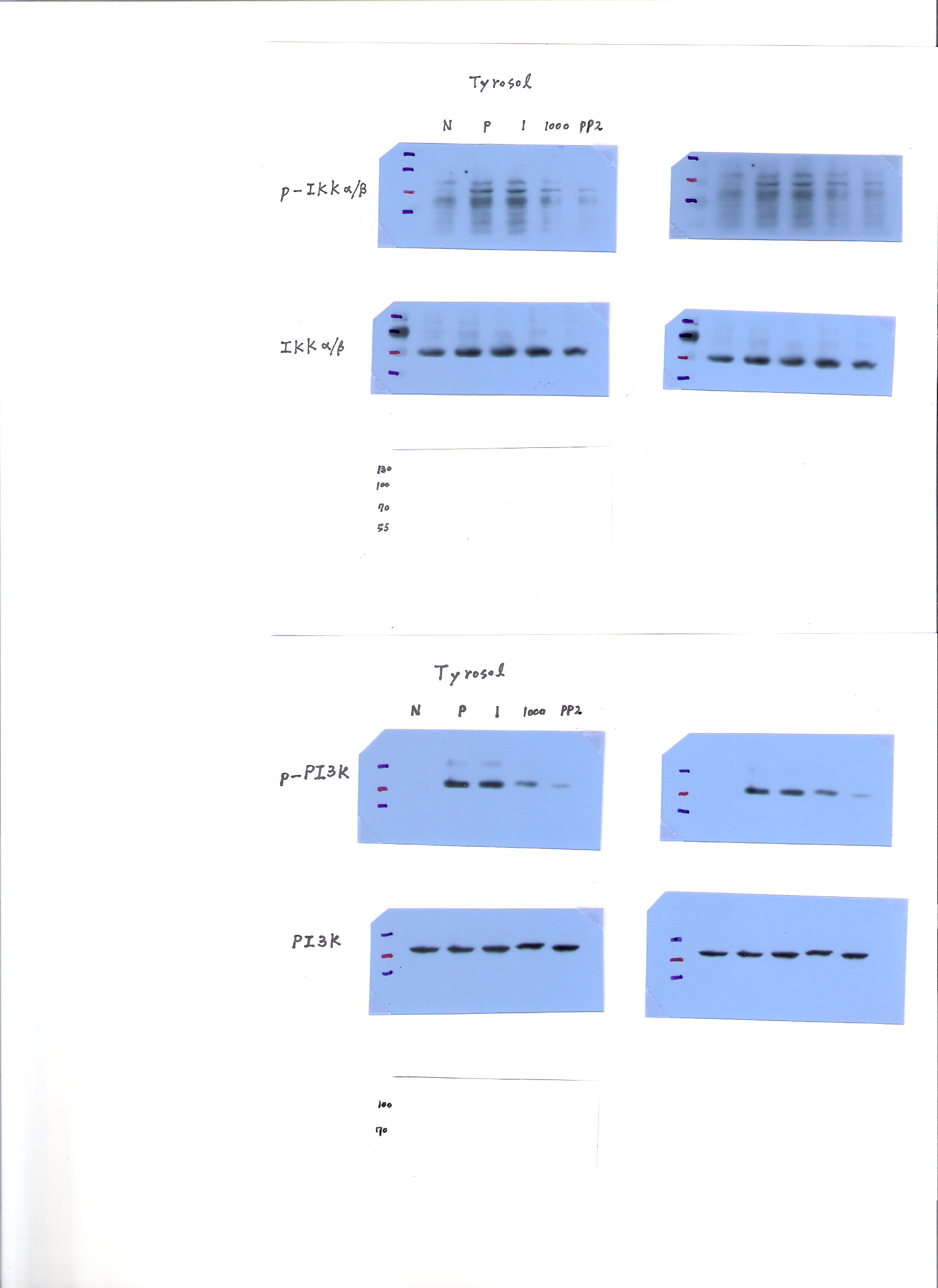

Supplement: S1 Archive — (ZIP) [file pone.0129829.s003.zip › Tyrosol PI3K, IKK.jpg]

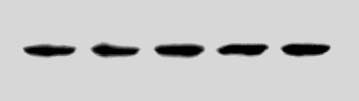

Supplement: S1 Archive — (ZIP) [file pone.0129829.s003.zip › Tyrosol PI3K.jpg]

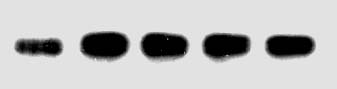

Supplement: S1 Archive — (ZIP) [file pone.0129829.s003.zip › Tyrosol Syk.jpg]

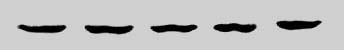

Supplement: S1 Archive — (ZIP) [file pone.0129829.s003.zip › Tyrosol actin.jpg]

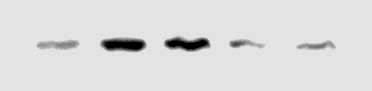

Supplement: S1 Archive — (ZIP) [file pone.0129829.s003.zip › Tyrosol p-Akt.jpg]

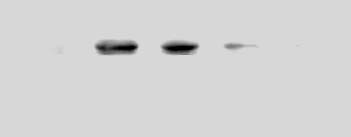

Supplement: S1 Archive — (ZIP) [file pone.0129829.s003.zip › Tyrosol p-IKK.jpg]

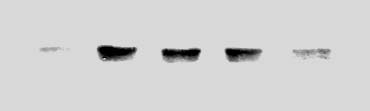

Supplement: S1 Archive — (ZIP) [file pone.0129829.s003.zip › Tyrosol p-Lyn.jpg]

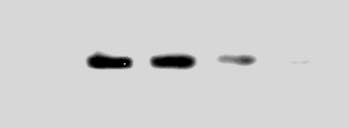

Supplement: S1 Archive — (ZIP) [file pone.0129829.s003.zip › Tyrosol p-PI3K.jpg]

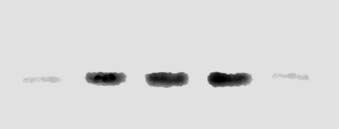

Supplement: S1 Archive — (ZIP) [file pone.0129829.s003.zip › Tyrosol p-Syk.jpg]
